# Clinical and biomarker factors affecting survival in patients with platinum-sensitive relapsed ovarian cancer receiving olaparib monotherapy: a multicenter retrospective study

**DOI:** 10.1038/s41598-023-39224-0

**Published:** 2023-07-24

**Authors:** Ryota Tashiro, Hitoshi Kawazoe, Kanako Mamishin, Keisuke Seto, Ryoko Udagawa, Yoshimasa Saito, Hironobu Hashimoto, Tatsunori Shimoi, Kan Yonemori, Masahito Yonemura, Hiroyuki Terakado, Takahiro Nishimura, Toshikatsu Kawasaki, Tetsuya Furukawa, Tomonori Nakamura

**Affiliations:** 1 Department of Pharmacy, National Cancer Center Hospital, Tokyo, Japan; 2 Division of Pharmaceutical Care Sciences, Keio University Graduate School of Pharmaceutical Sciences, Tokyo, Japan; 3 Division of Pharmaceutical Care Sciences, Center for Social Pharmacy and Pharmaceutical Care Sciences, Faculty of Pharmacy, Keio University, Tokyo, Japan; 4 Department of Pharmacy, National Cancer Center Hospital East, Chiba, Japan; 5 Department of Pharmacy, Center Hospital of the National Center for Global Health and Medicine, Tokyo, Japan; 6 Department of Medical Oncology, National Cancer Center Hospital, Tokyo, Japan

**Keywords:** Gynaecological cancer, Prognostic markers

## Abstract

The standard treatment for platinum-sensitive relapsed ovarian cancer (PSROC) is platinum-based chemotherapy followed by olaparib monotherapy. A retrospective study was conducted to identify factors affecting the survival of patients with PSROC undergoing olaparib monotherapy in real-world clinical settings. The study enrolled 122 patients who received olaparib monotherapy between April 2018 and December 2020 at three national centers in Japan. The study used the Kaplan–Meier method and univariable and multivariable Cox proportional hazards models to evaluate the associations between factors and progression-free survival (PFS). Patients with *BRCA1/2* mutations had a significantly longer median PFS than those without these mutations. Both the *BRCA1/2* mutation-positive and mutation-negative groups exhibited a prolonged PFS when the platinum-free interval (PFI) was ≥ 12 months. Cancer antigen 125 (CA-125) level within reference values was significantly linked to prolonged PFS, while a high platelet-to-lymphocyte ratio (≥ 210) was significantly associated with poor PFS in the *BRCA1/2* mutation-negative group. The study suggests that a PFI of ≥ 12 months may predict survival after olaparib monotherapy in patients with PSROC, regardless of their *BRCA1/2* mutation status. Additionally, a CA-125 level within reference values may be associated with extended survival in patients without *BRCA1/2* mutations. A larger prospective study should confirm these findings.

## Introduction

Ovarian cancer has a poor prognosis; after cervical and uterine cancers, it has the third-highest mortality rate worldwide among gynecological cancers^1^. The mechanism of action of olaparib entails inhibition of the poly (adenosine diphosphate-ribose) polymerase (PARP) enzyme and PARP trapping; this mechanism of treatment is different from that of conventional cytotoxic chemotherapies, such as platinum and taxanes^2^. Based on the results of a pivotal multinational phase III trial (SOLO-2 study) and results of a subgroup analysis of the phase II trial (Study-19), olaparib (a small-molecule targeted drug) has become the new standard maintenance therapy after a platinum regimen for patients with platinum-sensitive recurrent ovarian cancer (PSROC)^3,4^.

Olaparib traps PARP at sites of single-strand DNA breaks and inhibits its release, thereby blocking DNA repair and causing double-strand breaks. Double-strand breaks are not accurately repaired in a homologous recombination defect (HRD), as in tumors with breast cancer susceptibility (*BRCA1/2*) mutations; PARP inhibition in tumors with HRD results in tumor cell death via synthetic lethality^2^. Therefore, the *BRCA1/2* mutation-positive status is a predictive biomarker of a positive response to olaparib maintenance therapy in patients with PSROC. Furthermore, progression-free survival (PFS) after the initiation of olaparib monotherapy is reported to be longer in patients with PSROC and *BRCA1/2* mutations than in patients with PSROC without *BRCA1/2* mutations^4^.

However, PSROC and HRDs occur even without *BRCA1/2* mutations^5^. Moreover, *BRCA* gene testing is expensive, and a diagnosis of hereditary breast-ovarian cancer causes psychological stress in patients and their families. Thus, it is necessary to identify predictive factors of the response to olaparib maintenance therapy in patients with PSROC but without *BRCA1/2* mutations.

Platinum-free interval (PFI), cancer antigen 125 (CA-125) normalization after the last dose of the platinum regimen and response after the last dose of the platinum regimen have been suggested as new predictive factors of response to olaparib maintenance monotherapy in patients with *BRCA1/2* mutations and PSROC^6^. However, there are few reports on the predictors of response to olaparib in patients with recurrent *BRCA1/2* mutation-positive ovarian cancer, and the available evidence is insufficient^6^. The neutrophil-to-lymphocyte ratio (NLR) and the platelet-to-lymphocyte ratio (PLR), indicators of inflammatory response and immune function, have been reported as poor prognostic factors in primary and recurrent ovarian cancer^7,8^.

A phase II trial on patients with PSROC without *BRCA1/2* mutations revealed that compared with the placebo, olaparib markedly prolonged the PFS in those sensitive to previous platinum-based therapy^4^. Therefore, olaparib has been approved for maintenance treatment, without *BRCA* gene testing, in patients with PSROC who are sensitive to previous platinum-based therapy in Japan, Europe, and the United States. Recently, post-marketing surveillance showed that the PFS with olaparib maintenance monotherapy in patients with PSROC and the *BRCA1/2* mutation-negative status was comparable with that reported in a previous phase II trial^9^. To the best of our knowledge, however, no studies have explored the factors predicting a positive response to olaparib maintenance monotherapy in patients with PSROC and the *BRCA1/2* mutation-negative status.

Platinum-based drugs form DNA interstrand crosslinks, resulting in DNA double-strand breaks. Moreover, HRD tumors may be more susceptible to platinum-based therapy^10^. Therefore, we expect that platinum sensitivity-associated factors that are suggested to be predictors of olaparib efficacy in patients with PSROC and the *BRCA1/2* mutation-positive status would also apply to patients with PSROC and the *BRCA1/2* mutation-negative status.

We hypothesized that a combination of clinical factors and peripheral blood markers could help predict the clinical response to olaparib monotherapy in patients with PSROC, regardless of their *BRCA1/2* mutation status. Therefore, we performed an exploratory pilot study using real-world data to clarify the patient-associated clinical factors that affected survival in patients treated with olaparib monotherapy for PSROC with or without *BRCA1/2* mutations.

## Methods

### Study design and patients

This multicenter, retrospective, observational study was conducted at three high-volume centers, namely, the National Cancer Center Hospital (Tokyo, Japan), National Cancer Center Hospital East (Chiba, Japan), and Center Hospital of the National Center for Global Health and Medicine (Tokyo, Japan). The surveillance program at each facility is the same. Patient data were extracted from electronic medical records, and data integration and subsequent analyses were performed at the Keio University Graduate School of Pharmaceutical Sciences (Tokyo, Japan). The methodology adopted in this study followed the STROBE statement^11^.

The inclusion criteria were as follows: (1) consecutive patients aged ≥ 20 years who were diagnosed with PSROC and (2) patients who had received olaparib monotherapy (300 mg taken orally in tablet form twice daily) as maintenance between April 2018 and December 2020. The treatment schedule and follow-up were modified at the discretion of each clinician according to the efficacy and toxicity profile of each patient.

The exclusion criteria were as follows: (1) consent not provided for the use of medical records for research, (2) insufficient data from the patients’ medical records or lack of baseline laboratory data, (3) lower olaparib dosage at therapy initiation (100–250 mg taken orally twice daily), (4) received olaparib monotherapy once, discontinued olaparib for any reason, and then received olaparib monotherapy again after other chemotherapy, and (5) previously treated with bevacizumab or receiving olaparib and bevacizumab concomitantly.

The study protocol was approved by the Ethics Committees of the National Cancer Center (approval number: 2021-052) and Center Hospital of the National Center for Global Health and Medicine (approval number: NCGM-G-004274-00). The study was conducted in accordance with the principles of the Declaration of Helsinki and Ethical Guidelines for Medical and Health Research involving Human Subjects (Ministry of Education, Culture, Sports, Science, and Technology and Ministry of Health, Labour and Welfare; Japan). The need for written or oral informed consent was waived by the ethics review committees of the National Cancer Center and Center Hospital of the National Center for Global Health and Medicine owing to the retrospective nature of the study. Accordingly, we allowed patients to opt-out using the official website of each institution.

### Data collection

Patient data were de-identified and analyzed anonymously. We extracted the necessary baseline clinical and demographic data (last blood counts obtained within 4 weeks prior to treatment initiation and other pre-treatment data). The following data were collected: age, body mass index, cancer type, International Federation of Gynecology and Obstetrics staging, Eastern Cooperative Oncology Group performance status (ECOG PS), medical history of chemotherapy, treatment line, date of progression or death at the time of olaparib initiation, presence or absence of germline *BRCA1/2* mutations, PFI, CA-125 level, objective tumor response to the last platinum-based chemotherapy according to the Response Evaluation Criteria in Solid Tumors (RECIST) version 1.1^12^, and daily available peripheral blood data (including absolute neutrophil, lymphocyte, and platelet counts at baseline). The date of disease progression was defined as the date of the first incidence of disease progression identified on computed tomography scans using RECIST or during a clinical evaluation by each clinician. The PFI was defined as the interval between the completion date of first-line platinum-based chemotherapy and the date of the first relapse. Platinum-sensitive patients were defined as those with a PFI of ≥ 6 months. We calculated the baseline NLR and PLR using routinely available blood cell counts at pre-dose values on the day of olaparib induction. The NLR was calculated by dividing the absolute neutrophil count by the absolute lymphocyte count; the PLR was calculated by dividing the absolute platelet count by the absolute lymphocyte count. The follow-up period ended on March 31, 2021.

### Endpoints

The primary endpoint of this study comprised the clinical factors associated with survival after olaparib maintenance therapy. The effectiveness of treatment was evaluated using PFS and overall survival (OS). PFS was defined as the period from the date of olaparib treatment initiation to the date of disease progression or death from any cause. OS was defined as the period from the date of olaparib initiation to the date of death due to any cause. Patients without disease progression and those who survived were defined as censored to PFS and OS, respectively, on the date of the last follow-up.

### Statistical analyses

Patients were categorized into the *BRCA1/*2 mutation-positive and *BRCA1/*2 mutation-negative groups. Patient characteristics were summarized using descriptive statistics, including frequencies and proportions. The Kaplan–Meier method was used to estimate PFS and OS. The log-rank test was used to compare differences between survival curves. Receiver operating characteristic (ROC) curve analyses and the Youden’s index were used to determine the optimal cutoff values for age, NLR, and PLR associated with PFS^13^. In the ROC curve analyses, a larger area under the curve (AUC) indicated better predictive ability. Univariable and multivariable Cox proportional hazards models were used to evaluate the association between patient-associated clinical factors and survival endpoints. The proportional hazards assumption was not tested because this was not a prospective study. Hazard ratios (HRs) and 95% confidence intervals (CIs) are presented. Potential explanatory variables reported by several previous studies, specifically the PFI, baseline CA-125 level, and objective tumor response to the last platinum-based chemotherapy according to RECIST, were included as covariates in the univariable and multivariable models^3,4,6^. Furthermore, we conducted subset analyses according to the *BRCA1/2* mutation status, as they are part of the genes responsible for robust olaparib efficacy. We examined whether the clinical factors previously suggested as predictors of *BRCA1/2* mutations were applicable in this study^3,4,6^. In the *BRCA1/2* mutation-negative group, we also examined whether clinical factors related to platinum sensitivity were associated with survival endpoints. All statistical analyses were performed using JMP (version 16.2.0; SAS Institute Inc., Cary, NC, USA) and SPSS Statistics version 27 (IBM, Armonk, NY, USA). All *P*-values were two-sided, and *P*-values of < 0.05 indicated statistical significance.

### Ethics approval

This study was conducted in accordance with the principles of the Declaration of Helsinki and Ethical Guidelines for Medical and Health Research involving Human Subjects (Ministry of Education, Culture, Sports, Science, and Technology and Ministry of Health, Labour and Welfare; Japan). The study protocol was approved by the Ethics Committees of the National Cancer Center (approval number: 2021-052) and Center Hospital of the National Center for Global Health and Medicine (approval number: NCGM-G-004274-00).

### Consent to participate

The need for written or oral informed consent was waived by the ethics review committees owing to the retrospective nature of the study. Accordingly, we allowed patients to opt-out through the official website of each institution.

## Results

### Patient characteristics

**Figure 1:**
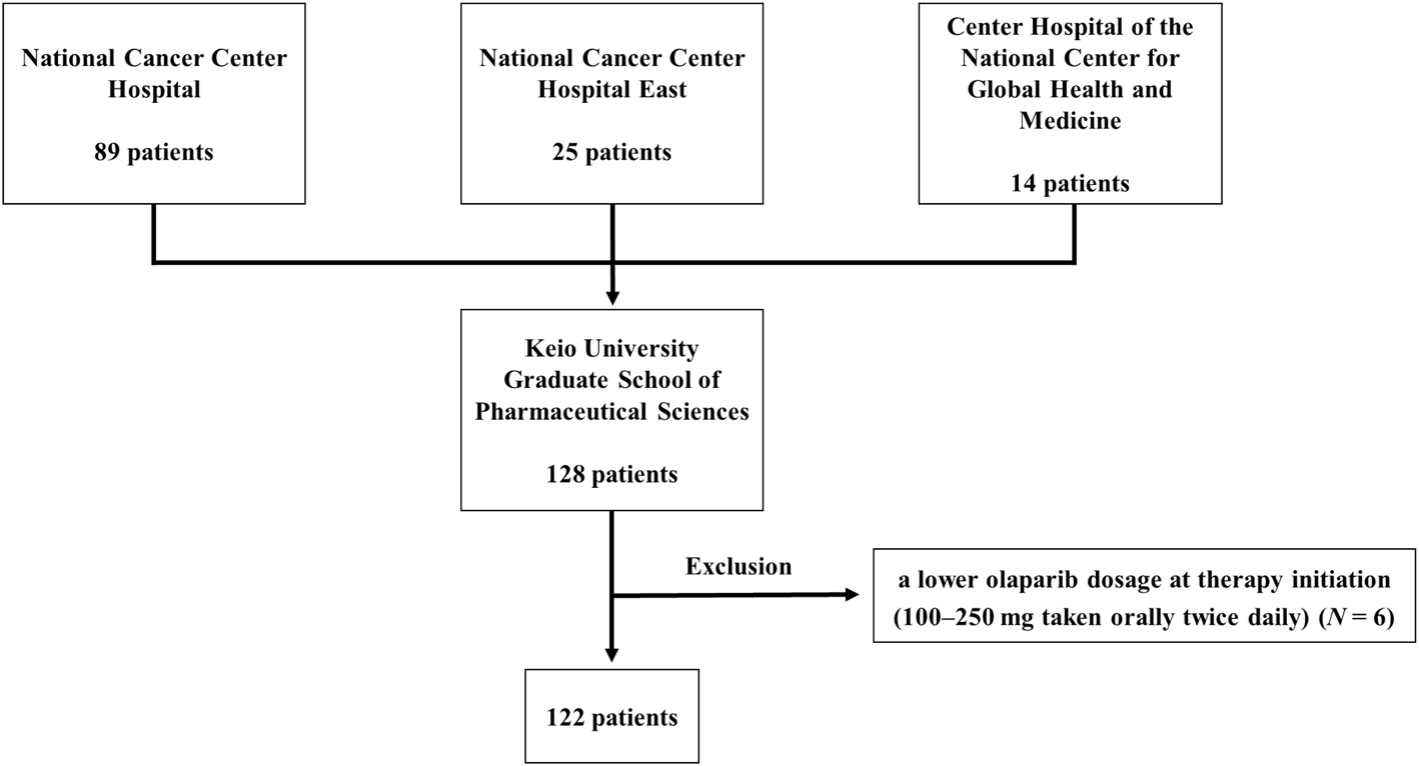
Flowchart illustrating the patient enrollment process.

The patient enrollment flowchart is shown in Fig. 1. Among the 128 patients initially identified, six were excluded based on the exclusion criteria; therefore, data from 122 patients were evaluated. The baseline demographic characteristics of these patients are listed in Table 1. The median age of the patients was 57 years (interquartile range [IQR], 49–68 years). Overall, 91 (74.6%) patients were in good condition with an ECOG PS of 0. The *BRCA1/2* mutation-positive group comprised 42 (34.4%) patients, while the *BRCA1/2* mutation-negative group comprised 61 (50.0%) patients. The median NLR and PLR were 1.9 (IQR, 1.5–2.5) and 172 (IQR, 139–214), respectively. All patients had a PFI of at least 6 months, and the response to the last platinum dose according to RECIST was either complete response or partial response, indicating that all patients in this study were platinum-sensitive and there were no non-response patients.

**Table 1:** Baseline demographic characteristics of the included patients.

Characteristics	All (*N* = 122)	*BRCA1/2* mutation			
		Positive (*N* = 42)		Negative (*N* = 61)	Unknown (*N* = 19)
Age (years), median (IQR)	57 (49–68)	56 (48–66)		62 (54–69)	53 (42–62)
< 60 years, *N* (%)	65 (53.3)	27 (64.3)		26 (42.6)	12 (63.2)
≥ 60 years, *N* (%)	57 (46.7)	15 (35.7)		35 (57.4)	7 (36.8)
FIGO stage, *N* (%)					
2	2 (1.6)	0 (0)		0 (0)	2 (10.5)
≥ 3	98 (80.3)	39 (92.9)		50 (82.0)	9 (47.4)
Unknown	22 (18.0)	3 (7.1)		11 (18.0)	8 (42.1)
ECOG PS, *N* (%)					
0	91 (74.6)	31 (73.8)		45 (73.8)	15 (78.9)
1–2	31 (25.4)	11 (26.2)		16 (26.2)	4 (21.1)
Histology type, *N* (%)					
Serous	115 (94.3)	40 (95.2)		57 (93.4)	18 (94.7)
Endometrioid	6 (4.9)	2 (4.8)		3 (4.9)	1 (5.3)
Others	1 (0.8)	0 (0)		1 (1.6)	0 (0)
Previous platinum regimens, *N* (%)					
≤ 2	118 (96.7)	41 (97.6)		58 (95.1)	19 (100)
> 2	4 (3.3)	1 (2.4)		3 (4.9)	0 (0)
PFI, *N* (%)					
6–12 months	62 (50.8)	22 (52.4)		30 (49.2)	10 (52.6)
≥ 12 months	60 (49.2)	20 (47.6)		31 (50.8)	9 (47.4)
CA125 level before olaparib treatment, *N* (%)					
≤ 35 U/mL	(93.2)	34 (81.0)		45 (73.8)	14 (73.7)
> 35 U/mL	29 (23.8)	8 (19.0)		16 (26.2)	5 (26.3)
Response to the last platinum dose according to RECIST					
Complete response, *N* (%)	20 (16.4)	7 (16.7)		11 (18.0)	2 (10.5)
Partial response, *N* (%)	102 (83.6)	35 (83.3)		50 (82.0)	17 (89.5)
NLR, median (IQR)	1.9 (1.5–2.5)	1.7 (1.3–2.2)		1.9 (1.5–2.7)	1.9 (1.5–2.1)
PLR, median (IQR)	172 (139–214)	170 (141–209)		176 (142–217)	178 (105–285)

*BRCA* breast cancer susceptibility gene, *IQR* interquartile range, *FIGO* International Federation of Gynecology and Obstetrics, *ECOG PS* Eastern Cooperative Oncology Group performance status, *PFI* platinum-free interval, *CA-125* cancer antigen 125, *RECIST* Response Evaluation Criteria in Solid Tumors, *NLR* neutrophil-to-lymphocyte ratio, *PLR* platelet-to-lymphocyte ratio.

### Endpoints

**Figure 2:**
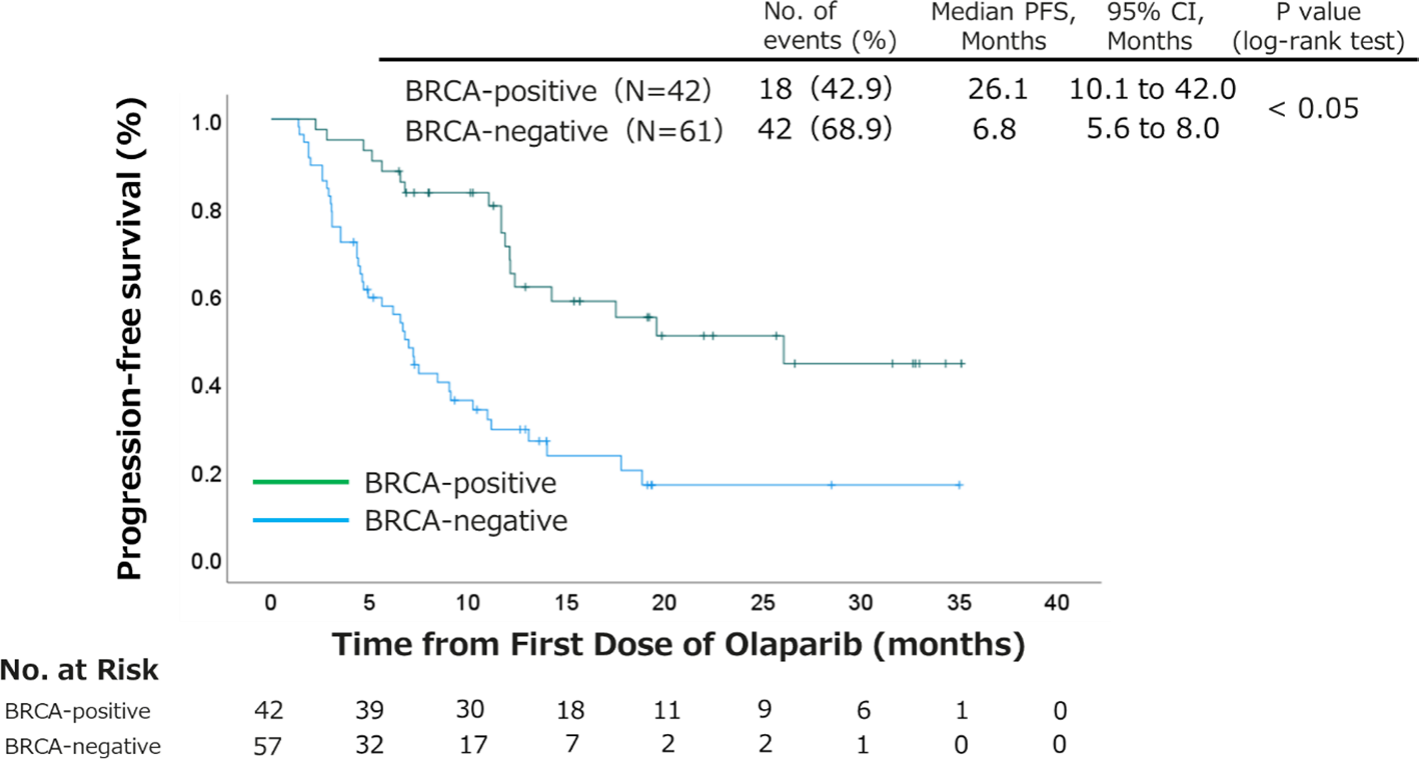
Kaplan–Meier survival curves for progression-free survival according to the *BRCA* mutation status-based subgroups. The log-rank test was used to compare the survival curves. *BRCA* breast cancer susceptibility gene, *CI* confidence interval, *PFS* progression-free survival.

The median follow-up period was 13.8 months (95% CI, 12.2–15.5 months). Overall, 73 progressive events and 31 deaths occurred. For all patients, the median PFS and OS were 11.0 months (95% CI, 8.2–13.8 months) and 33.5 months (95% CI, 29.6–37.4 months), respectively (Supplementary Fig. S1). Additionally, the median PFS in the *BRCA1/2* mutation-positive and *BRCA1/2* mutation-negative groups was 26.1 (95% CI, 10.1–42.0) and 6.8 (95% CI, 5.6–8.0) months, respectively; this difference was statistically significant (Fig. 2, *P* = 0.001).

The optimal cutoff values for age, NLR, and PLR for predicting the onset of progression at the median PFS were initially determined to be 63 years, 3.32, and 210, respectively, with corresponding Youden’s index values of 0.174, 0.101, and 0.199, respectively. The AUC values for age, NLR, and PLR were 0.581, 0.487, and 0.535, respectively. Therefore, we decided that an age of 60 years, NLR ≥ 3.32, and PLR ≥ 210 were appropriate cutoff values for further analyses.

The multivariable Cox proportional hazards model revealed the following as factors that were significantly associated with prolonged PFS (Table 2): a PFI of ≥ 12 months (adjusted HR, 0.38; 95% CI, 0.20–0.70; *P* = 0.003), CA-125 level within reference values (≤ 35 U/mL [adjusted HR, 0.46; 95% CI, 0.25–0.89; *P* = 0.016]), and *BRCA1/2* mutation-positive status (adjusted HR, 0.34; 95% CI, 0.18–0.61; *P* = 0.001). Conversely, a high PLR (≥ 210) was significantly associated with worse PFS among all patients (adjusted HR, 3.10; 95% CI, 1.60–5.83; *P* = 0.001), when adjusted for the selected covariates and the PFI, CA-125 level, PLR, and *BRCA1/2* mutation status.

**Table 2:** Patient-specific clinical factors associated with a prolonged progression-free survival among all patients (*N* = 122).

Variable	*N*	No. of events	(%)	Univariable analysis		Multivariable analysis	
				Crude HR (95% CI)	*P*-value	Adjusted HR (95% CI)	*P*-value
PFI							
≥ 12 months	60	35	58.3	0.48 (0.29–0.80)	0.005	0.38 (0.20–0.70)	0.003
< 12 months	62	38	61.3	1		1	
Response to the last platinum dose according to RECIST							
Complete	20	12	60.0	0.86 (0.38–1.72)	0.701		
Partial	102	61	59.8	1			
CA-125 level within reference values after the last platinum dose							
≤ 35 U/mL	93	51	54.8	0.40 (0.24–0.69)	0.001	0.46 (0.25–0.89)	0.016
> 35 U/mL	29	22	75.9	1		1	
Age							
≥ 60 years	57	32	56.1	0.98 (0.62–1.56)	0.945		
< 60 years	65	41	63.1	1			
NLR							
≥ 3.32	18	10	55.6	1.00 (0.44–1.97)	0.994		
< 3.32	104	63	60.6	1			
PLR							
≥ 210	37	24	64.9	1.98 (1.19–3.24)	0.007	3.10 (1.60–5.83)	0.001
< 210	85	49	57.6	1		1	

*HR* hazard ratio, *CI* confidence interval, *PFI* platinum-free interval, *RECIST* Response Evaluation Criteria in Solid Tumors, *CA-125* cancer antigen 125, *NLR* neutrophil-to-lymphocyte ratio, *PLR* platelet-to-lymphocyte ratio.

The multivariable Cox proportional hazards model revealed that a PFI of ≥ 12 months was significantly associated with prolonged PFS in patients with PSROC and the *BRCA1/2* mutation-positive status (adjusted HR, 0.32; 95% CI, 0.10–0.86; *P* = 0.033) (Table 3), when adjusted for the selected covariates and the PFI and PLR. In contrast, the multivariable Cox proportional hazards model revealed the following as factors that were significantly associated with prolonged PFS (Table 4): a PFI of ≥ 12 months (adjusted HR, 0.44; 95% CI, 0.20–0.89; *P* = 0.027) and CA-125 level within reference values (adjusted HR, 0.42; 95% CI, 0.20–0.96; *P* = 0.030). Conversely, a high PLR (≥ 210) was significantly associated with poor PFS in patients with PSROC and the *BRCA1/2* mutation-negative status (adjusted HR, 3.05; 95% CI, 1.38–6.58; *P* = 0.005), when adjusted for the selected covariates and the PFI, CA-125 level, and PLR. Owing to the small number of death events, a multivariable analysis for OS could not be performed.

**Table 3:** Patient-specific clinical factors associated with progression-free survival in the *BRCA1/2* mutation-positive group (*N* = 42).

Variable	*N*	No. of events	(%)	Univariable analysis		Multivariable analysis	
				Crude HR (95% CI)	*P*-value	Adjusted HR (95% CI)	*P*-value
PFI							
≥ 12 months	20	9	45.0	0.35 (0.11–0.94)	0.048	0.32 (0.10–0.86)	0.033
< 12 months	22	9	40.9	1		1	
Response to the last platinum dose according to RECIST							
Complete	7	3	42.9	2.15 (0.49–6.78)	0.239		
Partial	35	15	42.9	1			
CA-125 level within reference values after the last platinum dose							
≤ 35 U/mL	34	14	41.2	0.51 (0.18–1.82)	0.243		
> 35 U/mL	8	4	50.0	1			
Age							
≥ 60 years	15	7	46.7	1.10 (0.42–2.80)	0.839		
< 60 years	27	11	40.7	1			
NLR							
≥ 3.32	6	2	33.3	1.63 (0.37–5.02)	0.444		
< 3.32	36	16	44.4	1			
PLR							
≥ 210	12	8	66.7	2.52 (0.87–6.53)	0.066	2.35 (0.80–6.27)	0.096
< 210	30	10	33.3	1		1	

*BRCA* breast cancer susceptibility gene, *HR* hazard ratio, *CI* confidence interval, *PFI* platinum-free interval, *RECIST* Response Evaluation Criteria in Solid Tumors, *CA-125* cancer antigen 125, *NLR* neutrophil-to-lymphocyte ratio, *PLR* platelet-to-lymphocyte ratio.

**Table 4:** Patient-specific clinical factors associated with progression-free survival in the *BRCA1/2* mutation-negative group (*N* = 61).

Variable	*N*	No. of events	(%)	Univariable analysis		Multivariable analysis	
				Crude HR (95% CI)	*P*-value	Adjusted HR (95% CI)	*P*-value
PFI							
≥ 12 months	31	22	71.0	0.49 (0.24–0.95)	0.042	0.44 (0.20–0.89)	0.027
< 12 months	30	20	66.7	1		1	
Response to the last platinum dose according to RECIST							
Complete	11	8	72.7	0.46 (0.11–1.29)	0.202		
Partial	50	34	68.0	1			
CA-125 level within reference values after the last platinum dose							
≤ 35 U/mL	45	29	64.4	0.46 (0.24–0.95)	0.028	0.42 (0.20–0.96)	0.030
> 35 U/mL	16	13	81.3	1		1	
Age							
≥ 60 years	35	27	77.1	0.84 (0.45–1.57)	0.570		
< 60 years	26	15	57.7	1			
NLR							
≥ 3.32	10	6	60.0	0.77 (0.23–1.94)	0.623		
< 3.32	51	36	70.6	1			
PLR							
≥ 210	21	16	76.2	1.69 (0.85–3.26)	0.123	3.05 (1.38–6.58)	0.005
< 210	40	26	65.0	1		1	

*BRCA* breast cancer susceptibility gene, *HR* hazard ratio, *CI* confidence interval, *PFI* platinum-free interval, *RECIST* Response Evaluation Criteria in Solid Tumors, *CA-125* cancer antigen 125, *NLR* neutrophil-to-lymphocyte ratio, *PLR* platelet-to-lymphocyte ratio.

## Discussion

Although previous clinical trials have focused on prolonged survival with olaparib monotherapy, few studies have examined the clinical factors associated with survival in real-world clinical settings^6,14,15^. Furthermore, studies on clinical response biomarkers for olaparib monotherapy have included only patients with *BRCA1/2* mutations. The present study showed that a PFI of ≥ 12 months, CA-125 level within reference values, high PLR, and positive *BRCA1/2* mutation status might predict clinical response to olaparib monotherapy in all patients with PSROC. Additionally, a PFI of ≥ 12 months, CA-125 level within reference values, and high PLR may be associated with a prolonged PFS under maintenance therapy with olaparib in patients with PSROC and the *BRCA1/2* mutation-negative status. To the best of our knowledge, this is the first report on the clinical factors affecting survival following olaparib monotherapy in patients with PSROC with and without *BRCA1/2* mutations in a real-world setting.

Our findings are in line with those of a previous study, in which a PFI of ≥ 12 months was associated with prolonged PFS in patients with PSROC and *BRCA1/2* mutations^6^. Furthermore, previous studies on olaparib effectiveness in Chinese patients suggested that a PFI of ≥ 12 months and an objective tumor response to the last platinum-based chemotherapy (according to RECIST) could be considered clinical response biomarkers^14,15^. However, the associations between PFS and the objective tumor response to the last platinum-based chemotherapy (according to RECIST) and a CA-125 level within reference values were not significant in this study. This may be due to the small number of disease progression events.

Our findings may be explained by the mechanism of action of olaparib in ovarian cancer. Platinum drug-induced DNA double-strand breaks do not undergo repair in recurrent ovarian cancer cells with HRD; this leads to cell death. PFI is an indicator of platinum sensitivity, and a tumor with a PFI of ≥ 6 months is considered platinum-sensitive^3,4,6^. Objective tumor response (according to RECIST) and the CA-125 level are also used as clinical response biomarkers for anticancer drug efficacy. Olaparib inhibits DNA single-strand repair, and similar to platinum drugs, results in DNA double-strand breaks. Therefore, olaparib is thought to be effective in patients sensitive to platinum-based therapy.

In this study, the association between a high PLR and PFS was statistically significant, but that between a high NLR and PFS was not. Moreover, re-analyses of the relationship between baseline platelet and neutrophil counts and PFS revealed that only the platelet count was considerably associated with PFS. The platelet count is a poor prognostic factor for ovarian cancer^16^. Because no control group was included in this study, the baseline platelet count may have influenced the prognosis of the patients. Platelet-derived growth factors are present within the alpha granules of platelets, and the inhibition of platelet-derived growth factor receptors reduces *BRCA1/2* and *RAD51* expression, thereby decreasing homology-directed DNA repair^17^. This suggests that an increase in platelet count may have resulted in an increase in the platelet-derived growth factor levels, which in turn may have affected homology-directed DNA repair. However, no previous studies have shown the relationship between platelet counts and the effectiveness of olaparib monotherapy, and further cellular and clinical studies are needed on this.

The presence of *BRCA1/2* mutations is known to affect patient response to olaparib. However, patients with HRDs and the *BRCA1/2* mutation-negative status may also benefit from olaparib. Additionally, some patients have difficulty undergoing *BRCA* gene testing owing to the high cost of the test and the psychological stress associated with a hereditary breast and ovarian cancer diagnosis. Through subgroup analyses, phase II clinical trials overseas have shown that olaparib is effective in treating patients with PSROC and the *BRCA1/2* mutation-negative status, although the PFS after olaparib monotherapy initiation in these patients is shorter than that in patients with PSROC and the *BRCA1/2* mutation-positive status^4^. Moreover, patients who cannot undergo *BRCA* gene testing may also benefit from olaparib therapy, e.g., patients in Japan, who cannot undergo *BRCA1/2* somatic mutation testing or HRD testing under insurance coverage despite the fact that approximately 30% of all Japanese patients with recurrent ovarian cancer have *BRCA1/2* mutations and that somatic *BRCA1/2* mutations account for 5% of all patients with recurrent ovarian cancer overall. Subgroup analyses in phase II trials have suggested no differences in the efficacy of olaparib between patients with germline and somatic mutations^4^. In Japan, though comprehensive genomic profiling can be conducted to test for somatic *BRCA1/2* mutations, its wide application in clinical practice is hampered by its high cost and the reservation of testing in patients with primary ovarian cancer who cannot be treated with standard therapy. If insurance coverage changes, it may be possible to test for somatic *BRCA1/2* mutations.

This study has two strengths. First, this was a multicenter study that involved three national institutions with several patients with cancer in Japan. Therefore, our data may be generalizable to similar populations in clinical settings. Moreover, patient populations in randomized controlled trials differ from those encountered in daily practice. We believe that evidence from real-world data is important to support shared decision-making between clinicians and patients. Second, to the best of our knowledge, this is the first study to evaluate the clinical response biomarkers of olaparib monotherapy in patients with PSROC with or without *BRCA1/2* mutations.

This study has a few limitations. First, this was a retrospective, observational study; therefore, the effect of information bias cannot be ignored. However, we performed a multivariable analysis to reduce the influence of confounding factors related to observational studies and patient characteristics. Nevertheless, we could not control for confounders that were not measured in the multivariable analysis. Second, the sample size was relatively small; therefore, the number of progression events was insufficient. This may have influenced the difference in the detected associated factors between this study and the previous studies. In particular, the number of progression events in the *BRCA1/2* mutation-positive group was very small (18 cases). Thus, our findings should be validated by increasing the number of such cases. Third, owing to the retrospective nature of the study, we methodologically did not calculate the sample size. Fourth, the median follow-up period of 13.8 months was very short compared with the 37.3-month follow-up duration reported in a previous phase II trial^4^. This accounted for the small number of death events in this study; therefore, a multivariable analysis for OS could not be performed. Thus, investigations must be performed with a longer follow-up period in the future. Finally, our data was only olaparib. In Japan, olaparib and niraparib were approved in April 2018 and November 2020, respectively. Thus, we will validate niraparib in a future study.

The U.S. Food and Drug Administration has already limited the indication for PARP maintenance therapy for recurrent ovarian cancer to *BRCA1/2* mutation-positive cases, and we believe that it is important. On the other hand, this study was conducted with clinical factors that would be applicable to the patient population that cannot undergo *BRCA* mutation testing due to economic reasons or concerns about HBOCs. The results of this study suggest that a PFI of ≥ 12 months may be a clinical factor regardless of *BRCA* mutation.

In conclusion, our findings suggest that a PFI of ≥ 12 months may be associated with prolonged survival in patients with PSROC, irrespective of their *BRCA1/2* mutation status. Moreover, a PFI of ≥ 12 months and CA-125 level within reference values may be linked to prolonged survival in patients who have received olaparib monotherapy for PSROC and have the *BRCA1/2* mutation-negative status. Testing for these predictive factors is routinely accessible in clinical practice and can help classify and screen for high responders to olaparib monotherapy. Furthermore, the findings of this multicenter study can be extrapolated to the entire population of Japan.

## Supplementary Information

Supplementary Information.

## Data Availability

The data supporting the findings of this study are available on request from the corresponding authors after approval from the ethics committees. The data are not publicly available since they contain information that could compromise the patients’ privacy.
