# Supplementary material for: Clinical and biomarker factors affecting survival in patients with platinum-sensitive relapsed ovarian cancer receiving olaparib monotherapy: a multicenter retrospective study
Source: Sci Rep. 2023 Jul 24;13:11962. doi: 10.1038/s41598-023-39224-0 (PMC10366208; doi:10.1038/s41598-023-39224-0)
Supplement: Supplementary file 1 — Supplementary Information. [file 41598_2023_39224_MOESM1_ESM.docx]

Clinical and biomarker factors affecting survival in patients with platinum-sensitive relapsed ovarian cancer receiving olaparib monotherapy: A multicenter retrospective study

Ryota Tashiro, Hitoshi Kawazoe^*^, Kanako Mamishin, Keisuke Seto, Ryoko Udagawa, Yoshimasa Saito, Hironobu Hashimoto, Tatsunori Shimoi, Kan Yonemori, Masahito Yonemura, Hiroyuki Terakado, Takahiro Nishimura, Toshikatsu Kawasaki, Tetsuya Furukawa, Tomonori Nakamura

**(a)**

**
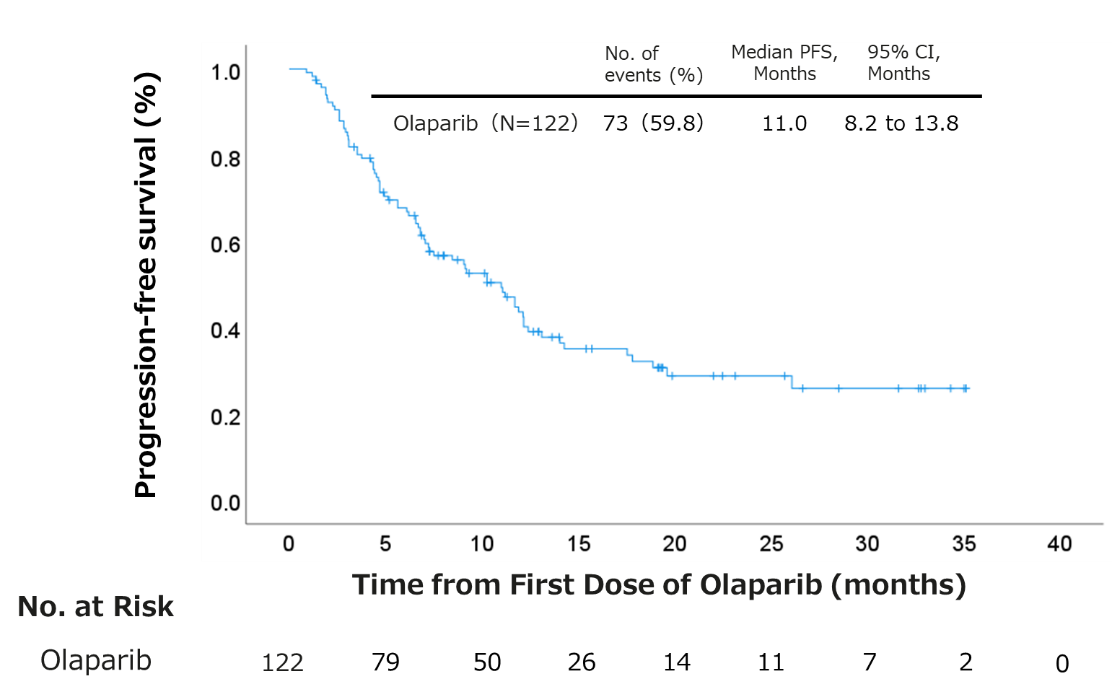
**

**(b)**

**
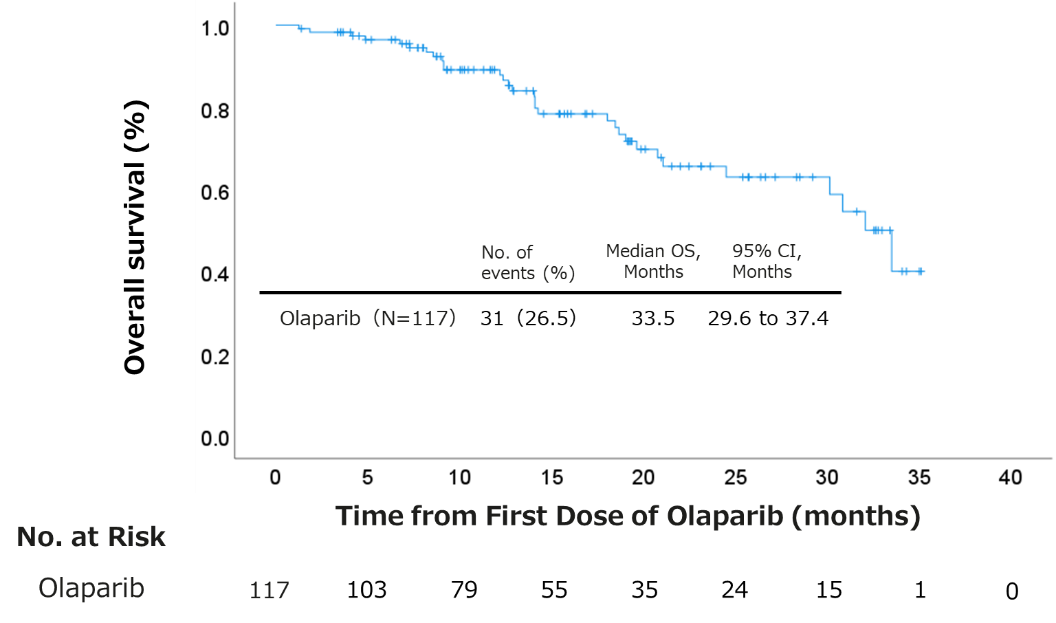
**

**Supplementary Fig. 1** Kaplan–Meier survival curves for progression-free survival and overall survival among all patients. (**a**) Progression-free survival. (**b**) Overall survival. Abbreviations: CI, confidence interval; OS, overall survival; PFS, progression-free survival.
